# SARS-CoV-2 pandemic as a catalyst? Development of emotional problems of preschool children of mothers with childhood maltreatment experiences in the course of the pandemic–a longitudinal analysis

**DOI:** 10.3389/fped.2023.1156282

**Published:** 2023-03-30

**Authors:** Manuela (Gulde) Dalhof, Katharina Rost, Ute Ziegenhain, Jörg M. Fegert, Franziska Köhler-Dauner

**Affiliations:** Department of Child and Adolescent Psychiatry/Psychotherapy, University of Ulm, Ulm, Germany

**Keywords:** children's emotional problems, SARS-CoV-2 pandemic, maternal childhood maltreatment (CM), risk factors, parental mental health

## Abstract

**Background:**

SARS-CoV-2 pandemic have posed great challenges for all families and children. Health risks and fears associated with SARS-CoV-2 negatively affect the parental mental health and perceived stress, which in turn influence parental coping and thereby impairs the mental health and well-being of their children. Additional risk factors within the parents, such as maternal childhood maltreatment (CM) experiences, may increase the risk of children to develop emotional problems during the pandemic.

**Objective:**

The purpose of this longitudinal study is to determine whether preschool children of mothers with CM are at higher risk of developing emotional problems during the pandemic than preschool children of mothers without CM.

**Method:**

74 mothers from a birth cohort examining pathways to resilience or vulnerability in the transgenerational transmission of CM, provided information on emotional problems of their children (aged 3–7 years) at two measurement time points (t1: May 2020, t2: March 2021) as part of an online “SARS-CoV-2 pandemic” survey. In addition, parents were asked for a retrospective assessment of their children's emotional problems before the pandemic at time t1. Children's emotional problems were assessed using the “emotional problems” scale of the German version of the Strengths and Difficulties Questionnaire (SDQ) and linked to previously collected data on mothers' childhood maltreatment experiences, which were collected using the German short version of the Trauma in Childhood Questionnaire (CTQ).

**Results:**

Our analyses showed that children's emotional problems increased significantly over the SARS-CoV-2 pandemic [*F*(1.86, 116.88) = 3.72, *p* = 0.030 *η*^2^ = 0.06] and were rated significantly higher in the group of children of mothers with CM, than in the group of mothers without CM [*F*(1, 63) = 126.06, *p* < 0.001 *η*^2 ^= 0.67]. Furthermore children's emotional problems of mothers with CM increased significantly more and reached a clinically significant value during the pandemic than for children of mothers without CM [*F*(1.86, 116.88) = 8.89, *p* < 0.001, *η*^2 ^= 0.12].

**Conclusions:**

Children of mothers with CM appear to be at increased risk of developing emotional problems during the pandemic. CM therefore needs to be considered as an additional risk factor in the impact of the pandemic on children.

## Introduction

Restriction in the context of SARS-CoV-2 pandemic and the loss of support by institutional social systems and other family members have posed great challenges for all families and children (1, 2). Within a few days, the lives of 13 million children and their families in Germany had changed abruptly. Schools and daycare centers were closed, playgrounds were off-limits, contact with friends and relatives was restricted, and the children and young people could no longer pursue their usual leisure activities (3), which led to a loss of daily routines and important developmental aspects (4).

Such epidemiologically required restrictions appear to be particularly stressful for families. Brooks and colleagues (5) point out that they endure a plethora of psychological distress, multiple neuropsychiatric manifestations, and psychosocial stigma (5).

Initial studies showed that the quality of life of children and adolescents in Germany declined during the pandemic due to changes and restrictions in their social life (6). Isolation, fear for grandparents, and lack of contact with friends have immediate and lasting psychological effects on children, as their lifestyle, physical activity levels, and mental activities change drastically (7, 8). Thus, current epidemiological restrictions have a demonstrable impact on the emotional and social development of children and adolescents.

Current research is investigating the effect of the ongoing pandemic on the mental health of children and adolescents. The longitudinal COPSY study (COrona and PSYche) of the University Medical Center Hamburg-Eppendorf (UKE) showed that emotional problems among children and adolescents increased from 16% to 24% during the pandemic. In addition, psychosomatic complaints in both children and adolescents increased significantly during the pandemic (e.g., abdominal pain from 21% before the pandemic to 36% during the second lockdown, dejection from 23% to 43%, headache from 28% to 46%, irritability from 40% to 57% and difficulty falling asleep from 39% to 47%) (3). Particularly children between 3 and 6 years old seem more likely to be affected by stress symptoms in their emotional and social development due to the pandemic (9). Findings at the beginning of the pandemic also indicate increased psychological distress for preschool-aged children (3–6 years) and a significant increase of behavioral problems and hyperactivity (4, 10). This finding is consistent with the study by Maldei-Göhring et al. (11), in which more than one-third of preschoolers also had psychological distress by the end of the second lockdown.

Preschool years have an important role in growth and adaption of a child's future. From the perspective of developmental psychology, preschool children go through a variety of developmental steps that require them to be able to experience and try out new situations and social interaction on a daily basis (12). As a developmentally vulnerable population, they need a stable and secure family environment, nurturing relationships, and emotional safety to cope with strains in times of stress and uncertainty, currently triggered primarily by the SARS-CoV-2 pandemic (13). Mentally healthy parents can be seen as an essential aspect here, who serve as a strong and protective factor in children's daily life (13).

Diverse studies found evidence that the health risks and fears associated with SARS-CoV-2 also negatively affect the parental mental health and perceived stress, which in turn influence parental coping and thereby impairs the mental health and well-being of their children (14–19). In particular parents of younger children showed a decrease in mental health since spring 2020 (20).

Several studies have already shown a strong association between parental and children's mental health under pre-pandemic normal conditions [e.g., (21–24)] and the role of caregiver well-being in supporting healthy parenting practices and positive material is also well established (25, 26). For example, Daud et al. (27) found an association between parents' traumatic history and their children's mental health: children of traumatized parents exhibited significantly more symptoms of anxiety, depression, posttraumatic stress, attention deficits, as well as conduct disorders (27). These findings are consistent with the recent research of Prime et al. (28) that also declared an association between preexisting vulnerabilities within the family, such as a parental history of psychological distress, and increased vulnerability to psychological distress in children. A recent meta-analysis of the intergenerational effects of maternal adverse childhood experiences (ACEs)—child abuse, neglect, and household dysfunction—found out that maternal childhood maltreatment had a significant impact on the offspring's depression and internalizing behaviors (29). By the age of 7 children of maltreated mothers were at an increased risk of clinically significant emotional and behavioral problems (30).

The very limited data available suggested a positive association between a history of maternal maltreatment in childhood and offspring mental health problems, including aggression, impulsivity, mood and anxiety disorders, posttraumatic stress disorder (PTSD), suicide attempts, and general emotional and behavioral problems, as well as poorer development of emotional adjustment over time (31–36). A link is hypothesized between maternal childhood maltreatment experiences and emotional problems in children, likely due to adverse parenting outcomes, for example parenting hostility, maternal emotional unavailability, and decreased maternal sensitivity (37, 38). Heleniak et al. (39) suggested a connection between childhood maltreatment experiences and increased emotional reactivity as a maladaptive response to stress, resulting in mothers with maltreatment childhood experiences experiencing more stress and less social support (39).

Thus, maternal experiences of childhood maltreatment could potentially act as a catalysator that significantly influences the impact of SARS-CoV-2 pandemic restrictions on children's mental health. As a developmentally vulnerable population that is particularly depending on family resources and regular social interactions for social-emotional development, this study hypothesizes that preschool children of mothers with childhood maltreatment experiences will be at higher risk for developing emotional problems during the pandemic than preschool children of mothers without childhood maltreatment experience.

## Methods

### Study design

The study TransGen was conducted through a cooperative interdisciplinary project with a prospective study design to investigate protective and risk factors concerning biological, psychological, and social components of the transgenerational transmission of maternal abuse. The joint project incorporates sub-projects, which consist of four clinical projects as well as an animal model and was financed by the Federal Ministry of Education and Research from October 2013 till March 2017. The Ethics Committee of Ulm University permitted the research project which got realized in concordance with requirements and policies.

The recruitment of mother-child-dyads took place during the first year after birth at the located maternity unit of the Ulm University. Using the German version of the Childhood Trauma Questionnaire (CTQ), maternal experiences of childhood maltreatment (CM) got assessed one to three days after the women gave birth (40, 41). This was supplemented by three follow-up measurements three months, twelve months, and three years postpartum. The data on maternal childhood maltreatment experiences used in the present study are from this first longitudinal study.

To measure the current stress level of the mothers as well as the children's emotional problems due to the pandemic two online “SARS-CoV-2 pandemic surveys” in two different time frames were offered. First, from May 18th–July 31st, 2020 after the first lockdown (t1), the second from March 1st–May 31st, 2021 after the second lockdown (t2). At the first survey time point (t1), parents were additionally asked for a retrospective assessment of their children's emotional problems before the pandemic (bp). To assess the emotional problems of preschool children the scale “emotional problems” of the German version of the Strengths and Difficulties Questionnaire [SDQ; (42)], a behavioral screening questionnaire which is filled in by a parent, was used.

The following results relate to the emotional problems of children over the time course of the pandemic.

### Participants

From October 2013 to December 2015 533 mother-child-dyads could be recruited for the study. Inclusion criteria for women compromised age ≥18 years, adequate use as well as understanding of the German language and, in addition, the health status of both mother and child. A woman's illness (e.g., AIDS disease, hepatitis, etc.), present/prior drug or alcohol abuse, acute mental health problems, severe birth complications, a premature birth with less than 37 weeks of pregnancy or a child with a vastly low weight of birth under 1,500 g depicted an exclusion criterion. For the first follow-up, including laboratory as well as home visits, collectively 240 mothers gave written informed consent, and were then asked to take part in the survey three months postpartum. The next measurement was realized about 12 months postpartum and included an additional laboratory and home visit with a total of 158 mother-child-dyads, followed by a third survey at the child´s age of three. All 158 mother-child-dyads were contacted again per mail and asked to participate in the additional online questionnaire “SARS-CoV-2 pandemic survey” concerning the effect of the pandemic on families on two independent measuring points (t1: May 18th–July 31st, 2020, t2: March 1st–May 31st, 2021). *N* = 91 of the contacted mothers were willing to edit the survey at t1 and *N* = 74 at t2. There were different reasons for not participating in measurement t1 and t2 like a lack of time, no willingness to take part in a particular survey concerning the SARS-CoV-2 pandemic or merely not reaching the families. Analysis was just executed for complete data sets on emotional problems of mother-child-dyads at both waves of data collection, resulting in *N* = 74 sets. Of the *N* = 17 participants from the first survey t1, no data on emotional problems were available at the second measurement point t2 for the reasons stated above and were therefore excluded.

### Measures

#### Consequences of SARS-CoV-2

In the “SARS-CoV-2-pandemic survey” numerous socio-demographic data of the mothers and their family were assessed. These included age, educational level and monthly income of the mother as well as number, age and gender of the children living in the household. Furthermore, changes in income and quantity of work due to the pandemic were surveyed and it was asked, whether they were currently working in a systemically relevant area (professional groups, which contribute to maintaining the economy, health system or basic services). Additional assessments of the online survey are not further explained because of its irrelevance for the present study.

#### Emotional problems of children

The children's emotional problems were assessed using the German version of the Strengths and Difficulties Questionnaire [SDQ; (42)], a behavioral screening questionnaire for children between 2 and 17 years old, which is filled in by a parent. With *r* = 0.7 the measurement exhibits an adequate Cronbach´s alpha (43). This instrument consists of five scales (emotional problems, externalizing behavioral problems, hyperactivity/attention problems, problems with peers and prosocial behavior) addressing positive and negative behavioral attributes of the children. Each scale contains 5 items and is rated on a 3-point Likert scale (0 = not applicable, 1 = partially applicable, 2 = clearly applicable). A total value can be calculated for all items as well as for each of the 5 scales, which provides information about the extent to which the behavior displayed is within the normal, borderline or conspicuous range.

For this study, only the scale “emotional problems” (Cronbach's alpha = 0.66) (44) at the measurement times t1 (May 18th–July 31st, 2020) and t2 (March 1st–May 31st, 2021) as well as the retrospective date assessed at t1 (bp) were analyzed.

For the emotional problems scale all five items were included: “frequently complains of headache, stomachache or nausea”, “has a lot of worries, often appears depressed”, “Often unhappy or depressed; often cries”, “nervous or clinging in new situations; easily loses self-confidence” and “has many fears; is easily afraid”. Sum values between 0 and 3 are in the normal range, the sum value of 4 marks the borderline range, and values between 5 and 10 are in the conspicuous range.

#### Maternal experiences of childhood maltreatment (CM)

Maternal childhood maltreatment experiences were assessed in a previous survey using the German short version of the Childhood Trauma Questionnaire (CTQ) (40, 41). The CTQ is a screening, retrospective self-report questionnaire for the assessment of child maltreatment. The CTQ contains five subscales each assessed by 5 items on a 5-point Likert scale, including emotional, physical and sexual abuse as well as physical and emotional neglect. Additionally, three items assess whether participants tend to trivialize problematic experiences. The psychometric properties of the German version of the CTQ have been demonstrated by Klinitzke and colleagues (45). The internal consistency range between 0.62 and 0.96 for the subscales. Severity scores for each subscale as well as a total score including all five subscales can be calculated, range from “none maltreatment experiences” (CM−) over “minimal” to “extreme” maltreatment load (CM+) (46). Mothers with a total score ≥6 were declared as CM+.

#### Statistical analysis

Data were analyzed using the Statistic Software R (Version 4.1.3). Statistical significance was set at *p* < 0.05 (two-tailed). Descriptive statistics were calculated to examine the variables' distributions and characteristics. *χ*^2^ tests were calculated to test the distribution of categorical variables in the sample.

We reviewed the bivariate association between potential control and key study variables ahead of our main analyses by calculating Pearson correlations, one-way-ANOVAS and two-sample-*t*-tests.

Inferential analyses were conducted as follows: a mixed analysis of variance (mixed ANOVA) with children's emotional problems (EP) as the dependent variable, time as the within-subject-factor, parental childhood maltreatment (CM) as the between-subject-factor and mother's education and children's sex as covariates were calculated. The requirements normality and homogeneity of variances for a mixed ANOVA were not met. In addition, the data set contained *n* = 20 extreme outliers on the variable emotional problems. Therefore, an additional robust ANOVA with 20% trimmed means was calculates in R using the “WRS2”-package.

## Results

### Descriptive analysis

Complete data of all measurements could be collected for *N* = 74 children (39 boys and 35 girls) and *N* = 74 mothers. 28 (38%) of the 74 mothers reported CM and were classified as CM+.

During the collection of the “SARS-CoV-2-pandemic-survey” the mother's age ranged from 32-to-50 years (*M* = 40.00, SD = 3.87), children's age ranged from 3-to-6 years before the pandemic (retrospective assessed at t1) (*M* = 4.45, SD = 0.62), 4-to-6 years at t1 (*M* = 5.12, SD = 0.70) and 5-to-7 years at t2 (*M* = 5.89, SD = 0.69). Boys and girls were almost equally represented with a slight overhang of boys (53%). Most women occupy a higher level of education with 54% having a university degree, 7% completed 13 years and 16% 10 years of school education (secondary school degree). Just 4% of the mothers attended 9 years or less at school. Concerning the COVID-19-pandemic 4% of the participants reported a reduction in income and another 8% a reduction of working hours. Descriptive data and results of *χ*^2^-tests are illustrated in Table 1.

**Table 1:** Descriptive data and results of *χ*^2^-tests.

	M	SD	Median	Min	Max
Mother's age	40.00	3.87	40	32	50
Children's age bp	4.45	0.62	4	3	6
Children's age at t1	5.12	0.70	5	4	6
Children's age at t2	5.89	0.69	6	5	7
** **	***N***	**%**	***χ* ^2^**	**Df**	***P***
CM					
Yes	28	38	4.38	1	.036
No	46	62			
Children's sex			0.22	1	.642
Male	39	53			
Female	35	47			
Education			82.63	4	<.001
University	42	57			
13 years	6	8			
10 years	13	18			
≤9 years	6	8			
Monthly income			32.38	6	<.001
>4,000 €	22	30			
3,500–4,000 €	13	18			
3,000–3,500 €	26	22			
2,500–3,000 €	11	15			
2,000–2,500 €	8	11			
1,500–2,000 €	1	1			
<1,500 €	2	3			
Diminish in income			49.60	1	<.001
Yes	3	4			
No	58	78			
Reduction working hours			32.67	1	<.001
Yes	6	8			
No	48	65			

Percentages do not add up to 100% due to missing data in some variables. bp, before pandemic (retrospective assessed at t1).

The mean score of children's emotional problems (EP) rated by their parents was *M* = 1.50 (SD = 1.58) before the pandemic (retrospective assessed at t1), *M* = 2.66 (SD = 1.67) at t1 and *M* = 3.27 (SD = 2.00) at t2 (Table 2). Descriptive data and results of *χ*^2^-tests are illustrated in Table 1.

**Table 2:** Descriptive data and correlations between all relevant variables.

Variable	M	SD	1	2	3	4	5	6	7
1 EP bp	1.50	1.58							
2 EP at t1	2.66	1.67	.77***						
3 EP at t2	3.27	2.00	.63***	.85***					
4 Mother's age	40.00	3.87	.10	.08	.01				
5 children's age bp	4.45	0.62	.13	.08	.10	.25*			
6 children's age t1	5.12	0.70	−0.01	−0.05	.00	.29*	.75***		
7 children's age t2	5.89	0.69	.00	−0.08	−0.06	.26*	.72***	.82***	

bp, before pandemic (retrospective assessed at t1).

* *p *< 0.05.

*** *p* < 0.001.

### Correlation analysis

The calculation of Pearson correlations showed a high intra-individual stability of emotional problems over all three measuring points (bp and t0: *r* = 0.77, *p* < 0.001, bp and t1: *r* = 0.63, *p* < 0.001, t0 and t1: *r* = 0.85, *p* < 0.001).

Mother's age, children's age, mother's highest education and children's sex were tested as covariates. Pearson correlations revealed no significance between mother's age and children's age with emotional problems at every measuring point (Table 2) and were therefore not included as covariates. Mother's highest education (retrospective (bp): *F*(3, 63) = 3.81, *p* = 0.014); t1: *F*(3, 63) = 5.97, *p* = 0.001) and children's sex [t1: *t*(67.97) = −1.09, *p* = 0.003]; t2: *t*(72) = −3.43, *p* = 0.001) had a significant effect on children's emotional problems at more than one measuring point and were therefore included as covariates.

### Emotional problems in children and maternal CM during the pandemic

A mixed ANOVA with children's sex and mother's highest education as covariates was calculated. Note, that homoscedasticity was not fulfilled for this analysis, results could therefore be distorted and should be interpreted with caution. The analysis showed, that both main effects, as well as the interaction effect were significant. Children's emotional problems increased significantly over time [*F*(1.86, 116.88) = 3.72, *p* = 0.030, *η*^2 ^= 0.06] and were rated significantly higher in the group with CM+ mothers, than in the group with CM− mothers [*F*(1, 63) = 126.06, *p* < 0.001, *η*^2 ^= 0.67]. Furthermore, the analysis showed, that children's emotional problems of children with CM+ mothers increased significantly more, than for children of CM− mothers [*F*(1.86, 116.88) = 8.89, *p* < 0.001, *η^2^*^ ^= 0.12].

Due to unmet preconditions and limited interpretability, a second analysis without covariates was calculated using a robust ANOVA with 20% trimmed means. The analysis showed, that both main effects, as well as the interaction effect were significant. Children's emotional problems increased significantly over time [*F*(2, 44.02) = 46.94, *p* < 0.001, *η^2^*^ ^= 0.12] and were rated significantly higher in the group with CM+ mothers, than in the group with CM− mothers [*F*(1, 42.02) = 126,06, *p* < 0.001, *η^2^*^ ^= 0.75]. Furthermore, the analysis showed, that children's emotional problems of mothers with CM increased significantly more, than for children with CM− mothers [*F*(2, 44.02) = 6.95, *p* = 0.002, *η^2^*^ ^= 0.24].

**Figure 1:**
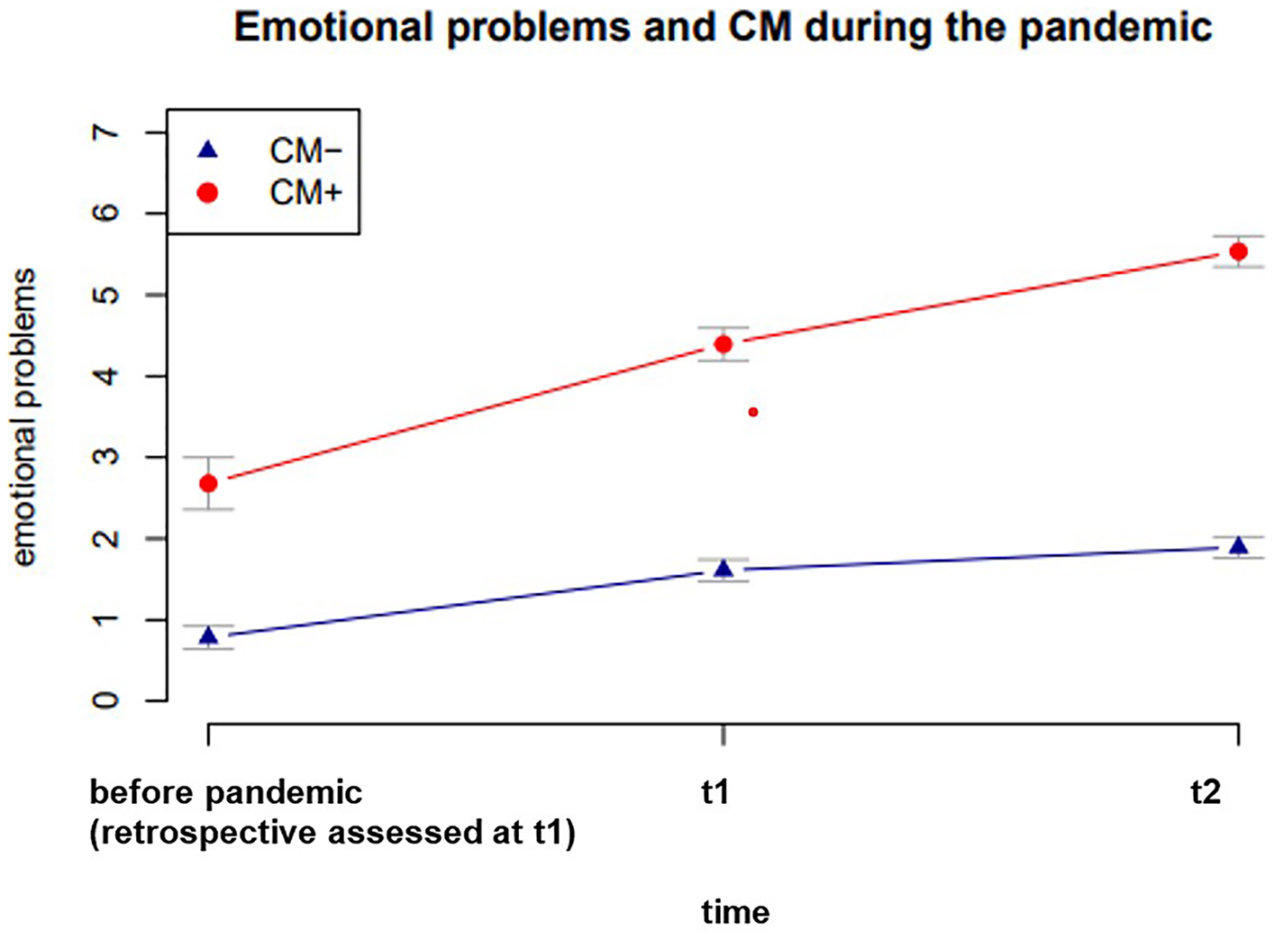
Means of children’s emotional problems as a function of CM and the SARS-CoV-2-pandemic with standard errors.

More importantly, the measured scores in the SDQ scale “emotional problems” of children with CM+ mothers reached at t1 a value in the borderline range: 4.39 (sum value of 4) and at t2 a value in the conspicuous range to clinically significant psychological distress: 5.54 (sum values ≥5) (see Table 3). Descriptive data of children's emotional problems at every measuring point with CM+ and CM− mothers is illustrated in Table 3 and Figure 1.

**Table 3:** Descriptive data M(SD) of children's emotional problems at every measuring point with CM+ and CM− mothers.

	CM−	CM+
Bp	0.78 (0.96)	2.68 (1.70)
t1	1.61 (0.91)	4.39 (1.07)
t2	1.89 (0.88)	5.54 (1.00)

bp, before pandemic (retrospective assessed at t1).

## Discussion

The aim of this study was to investigate if preschool children of mothers with childhood maltreatment experiences are at higher risk to develop emotional problems during the SARS-CoV-2 pandemic than preschool children of mothers without childhood maltreatment experiences.

First, we could demonstrate that emotional problems of all preschool children in the sample increased significantly over the course of the pandemic (see Table 3, Figure 1). This finding is consistent with current studies (2–4, 9–11). Our analyses showed that emotional problems of children of CM+ mothers were assessed significantly higher than in the group of children with CM− mothers and that emotional problems of children with CM+ mothers increased significantly more and reached a clinically significant value during the pandemic than those of children with CM− mothers. A high intra-individual stability of emotional problems over all three time points could be observed (see Table 3, Figure 1).

Several studies can be used to explain this effect. Children's developmental risk is related to caregivers' current mental and physical health status through multiple social and biological pathways, including epigenetic changes related to early adversity such as childhood maltreatment experiences (47–49). Numerous strands of research suggested that the experience of childhood maltreatment leads to permanent disruption of stress regulation abilities, negatively affecting both structures and functions of brain areas involved in mental health and the ability to regulate emotion and behaviour (50–60). Several research findings suggested that the impact of a maternal history of childhood maltreatment on one's children is dependent on the child's developmental status as well as ecosystem-level risk factors, i.e., environmental elements that have been shown to influence quality of care, including maternal exposure to stress/negative life events and access to social support (59). For example, the risk for poor care and/or child maltreatment among mothers with childhood maltreatment experiences may increase during the preschool years when the child is struggling for autonomy, testing limits, and having frequent and intense emotional outbursts (51). Maltreated mothers may respond inappropriately to these demands due to their own impaired self-regulatory abilities (51).

Disruption of stress regulation abilities can also impact the caregiving and interactional context in which it negatively affects maternal mental health and quality of care, and directly exposes the child to adverse growth conditions (36, 52). Several studies have demonstrated associations between a maternal childhood maltreatment history and insensitive caregiving behaviors, including hostility, harsh discipline, intrusiveness, inconsistency, lower involvement, and rejection (32, 34, 36, 53, 54). Exposure to such caregiving behaviors increases the risk for child's mental health problems (60). A few studies suggested that disrupted maternal caregiving partially mediates the association between maternal childhood maltreatment history and offspring mental health [e.g., (32, 36)], whereas other studies have found no such evidence (34).

Very limited data suggested that children of maltreated mothers experience greater psychosocial adversity and negative life events (32, 34). For example, Collishaw et al. (2007) (32) found out that children of mothers with childhood maltreatment experiences were not only exposed to a wide range of stressful life experiences in early childhood, but were also faced with an increasing number of different stressors between the ages of four and seven (32), and that these stressors partially mediate the association between maternal childhood maltreatment experiences and child's mental health problems (32, 34).

Because only few studies to date have examined the factors that influence the impact of maternal childhood maltreatment experiences on children's mental health, again, only hypotheses can be made about the individual mechanisms by which maternal childhood maltreatment experiences affect preschool children's mental health during the SARS-CoV-2 pandemic. The pandemic can be seen as a critical and stressful life event for both children and adults, which poses special challenges to both children and adults and demands a high level of stress regulation skills. If parents are limited in their stress regulation abilities as well as parenting skills due to their own childhood experiences, it can be assumed that mothers with CM+ will not be able to meet the needs of their children to the required extent in times of the pandemic and emotional problems in these children may increase or intensify during the pandemic, as our results showed.

However, the results do not allow to draw any conclusions about the individual mechanisms how maternal childhood maltreatment experiences acts on the mental health of preschool children in the context of the pandemic. It is unclear, for example, whether the children's emotional problems can be attributed solely to the parents' inability to regulate stress and meet the children's special needs during the pandemic, or whether these children are already limited in their own ability to regulate stress through transgenerational transmission or because they have already been exposed to increased stressors in their life history. Further research is needed to specify the link between maternal childhood maltreatment experiences and children's mental health in the context of the pandemic.

## Limitations

Considering the present study, we have to contemplate some limitations.

First, the collected data sets and online survey consist of a restricted sample of mother-child dyads of one mother birth cohort. Willingness to talk openly about their children's mental health could influence their consent to participate in the survey. In addition, it has to be taken into account that social desirability might impact the motheŕs evaluation of such questions. In addition, there may have been recall bias or underestimation by self-report, since children' s emotional problems before the pandemic, which was used as a baseline in this study, were recalled retrospectively by the mothers.

Due to the small sample size and the short survey duration during an early stage of the pandemic, future studies need to focus on the validation of our results and support our hypothesis with a representative sample size.

Secondly, our study was conducted at the end of the 1st and 2nd lockdown in Germany. Preliminary findings suggest that the burden in the population has decreased again since then (62). Therefore, to assess the long-term impact of the COVID-19 pandemic on children's mental health, a longitudinal analysis is needed. In this, additional protective and risk factors, such as parental mental health (63) as well as parental coping and parenting (64, 65) should be assessed to uncover critical evidence of mechanisms for child well-being and provide an empirical basis for implementing pandemic prevention programs.

Third, it has to be considered that a great number of our participants had a high standard of education, a partnership and did not suffer from reduction of income as a consequence of the pandemic. Thus it is difficult to generalize our results to all families or the general population. According to Meng et al., (66) psychological well-being and life satisfaction are influenced by education and socioeconomic status with an increase resulting in greater well-being and life satisfaction. Thus, it must be assumed that the emotional problems of children from families with low socioeconomic status could be even higher than in our present sample.

It should also be noted that the internal consistency of the emotional problems scale of the SDQ is low, with a value of Cronbach's alpha = 0.66 (44). Comparisons with existing studies on the development of emotional problems in preschool children during the pandemic, which were not conducted with the same measurement instrument (emotional problem scale of SDQ), should be made with caution.

Finally, there are some limitations regarding statistical analyses. A mixed ANOVA with children's sex and mother's highest education as covariates was calculated. Note, that Homoscedasticity was not fulfilled for this analysis, results could therefore be distorted and should be interpreted with caution. Due to unmet preconditions and limited interpretability, a second analysis without covariates was calculated using a robust ANOVA with 20% trimmed means. Both analyses showed, that both main effects, as well as the interaction effect were significant, so it can be assumed, that the results are statistically significant.

## Conclusion

Our study demonstrated that preschool children of mothers with childhood maltreatment experiences are at significantly higher risk of developing clinically significant emotional problems over the course of the pandemic.

Several studies have already addressed variables or family risk factors that influence children's mental health or led to psychological distress in the context of SARS-Cov-2- pandemic restrictions (e.g., low socioeconomic status (4, 6, 66), tight housing, preexisting mental illness, immigrant background, parents with a low level of education or who suffer from mental illness (3, 4, 67–71), age of the child, reduce income, dissatisfaction with shared childcare as consequences of the pandemic (64)).

Our findings show that maternal childhood experiences (CM+) also represent a risk factor for preschoolers' mental health that is amplified by pandemic conditions. CM+ therefore needs to be considered as an additional risk factor, that influences children's emotional well-being during the pandemic. Thus, the study makes an important contribution to the analysis of family risk factors affecting children's mental health during the pandemic and joins the research strand on risk factors.

Our findings suggest that pandemic disasters and subsequent containment efforts create a condition that can negatively affect the emotional health of young children and their mothers. Because of the increased dependence of children on their parents for stress regulation and the influence of parental stress on children's mental health, special response strategies are needed to address the emotional health needs of young children and their families. Pandemic mitigation measures must take these needs into account. Because pandemic disasters are unique and there are no held-forward interventions for prolonged support and recovery our findings reinforce existing calls [e.g., (3, 67, 69)] to expand preventive services to promote and maintain stress coping skills for both children and parents in order to maintain children's mental health in times of crisis.

## Data Availability

The original contributions presented in the study are includes in the article/supplementary material, further inquiries can be directed to the corresponding author.
